# IKK-mediated CYLD phosphorylation and cellular redox activity

**DOI:** 10.1186/s10020-022-00439-y

**Published:** 2022-02-02

**Authors:** Adnan Erol

**Affiliations:** Silivri-Istanbul, Turkey

**Keywords:** CYLD, IKK, NRF2, NF-κB, RIPK1

## Abstract

Oxidative stress is important in the development of obesity-related nephropathy (ORN). A causal relationship between IKK and ORN via CYLD-mediated inhibition of NRF2 has been described. However, contradictory explanations about the functioning of the mechanisms that will be effective in the pathogenesis require clarification.

In a recent study published in the Journal, Chen et al. claimed that IKKs, NF-κB activating kinases, may reduce the cellular antioxidant capacity in obesity-related nephropathy (ORN) through inactivating one of the major deubiquitinases, CYLD (Chen et al. 2021). According to the authors of the study, ox-LDL-stimulated oxidative stress may activate IKK that, in turn, phosphorylates and inactivates the deubiquitinating activity of CYLD. Consequently, the increased ubiquitination and degradation of NRF2 may promote oxidative stress injury in ORN cells. Importantly, they found that NRF2 is molecularly encapsulated by “a series of high-molecular-weight species” and directly binds CYLD. Furthermore, the treatment of ORN cells with MG132, a proteasome inhibitor, resulted in the activation of NRF2-related transcription. Altogether, they concluded that IKK-mediated inactivation of CYLD may potentiate the oxidative stress injury through the increased NRF2 degradation.

Proteasomal degradation of NRF2 will reduce the antioxidant ability of the cell. However, concluding that CYLD will protect NRF2 degradation by reducing its polyubiquitination would be controversial. On the contrary, a previous study reported that CYLD may increase oxidative stress by inhibiting the anti-oxidative activity of NRF2, as they cited in their study (Wang et al. 2015). CYLD, having deubiquitinase (DUB) activity, is a tumor suppressor that plays a key role in proliferation and cell death. CYLD negatively regulates the NF-κB signaling pathway by removing selectively Lysine (K)-63–linked and linear polyubiquitin chains but exhibits very little activity towards degradative K48-linked ubiquitin chains (Komander et al. 2009; Sato et al. 2015).

Previous studies with the tumor suppressor CYLD have described it as having an anti-inflammatory function, primarily because of its inhibitory effect on the NF-κB pathway. The Inhibition of CYLD increases resistance to apoptosis by activating NF-κB, suggesting a mechanism through which loss of CYLD contributes to oncogenesis (Brummelkamp et al. 2003). Furthermore, CYLD is believed to promote apoptosis and programmed necrosis (necroptosis) by facilitating RIPK1 deubiquitination (Moquin et al. 2013). RIPK1 is a complex protein that possesses both a scaffolding pro-survival as well as a catalytic pro-death function. When RIPK1 is modified by K63- and M1-linked ubiquitins, it acts as a scaffold, independent of its kinase activity, to recruit some adaptor proteins, leading to the NF-κB-dependent transcription of pro-survival proteins (Li et al. 2020). Furthermore, the IKK complex that phosphorylates CYLD is also necessary for the induction of NF-κB-dependent transcription. Until very recently, canonical IKK members, IKKα and IKKβ, and non-canonical IKKε were believed to phosphorylate and inactivate the DUB activity of CYLD (Reiley et al. 2005; Hutti et al. 2009). However, a very recent study made the issue more complicated. The research revealed that IKK phosphorylation, paradoxically, stimulates rather than inhibits CYLD, promoting its DUB activity (Elliot et al. 2021). Thus, IKK-mediated CYLD phosphorylation deconjugates ubiquitin chains on RIPK1, increasing its kinase activity for the death signaling pathway. Phosphorylated CYLD also reduces resistance to apoptosis by the loss of scaffolding function of RIPK1 necessary to activate NF-κB-mediated transcription.

NRF2, the main regulator of the cytoprotective gene program, is a transcription factor that encodes not only antioxidant genes but also numerous detoxification enzymes that conjugate oxidation products. Canonically, KEAP1, an adaptor component of ubiquitin E3 ligase, constitutively ubiquitinates NRF2 with K48-linked polyubiquitin chains. Consequently, KEAP1 promotes the proteasomal degradation of NRF2, thus keeping cellular NRF2 at a low level (Taguchi et al. 2012). On the other hand, in a noncanonical pathway, stimulated p62 can also bind to KEAP1. p62 is a stress-inducible protein, which serves as an adaptor protein between selective autophagy and ubiquitin signaling. In the non-canonical KEAP1-NRF2 pathway, mTORC1-mediated phosphorylation of p62 at serine-349 leads to the competitive binding with KEAP1. Following KEAP1 association with p62 and KEAP1-NRF2 dissociation, stabilized NRF2 translocates to the nucleus to induce its target genes. NRF2 positively regulates p62 gene expression; therefore, p62 is able to set up a positive feedback loop to activate NRF2, which in turn stimulates increased transcription of the p62 gene (Taguchi et al. 2012; Ichimura et al. 2013).

In the light of these data, it would be more rational to develop a new model between IKK-mediated CYLD phosphorylation and NRF2 activity. Under basal (unstimulated) conditions, KEAP1 constitutively ubiquitinates NRF2, leading to the rapid proteasomal degradation of NRF2 in the canonical KEAP1-NRF2 pathway (Fig. 1A). Following stimulation, CYLD undergoes phosphorylation at serine-418 and serine-568 residues catalyzed by either canonical IKKs (IKKα and IKKβ) or by the noncanonical IKKε, which may increase DUB activity of CYLD. The stimulated CYLD blocks the activation of mTORC1, increasing autophagy (Colombo et al. 2021). Consequently, the loss of phosphorylation of p62 by mTORC1 inhibits the interaction of p62 with KEAP1. Released KEAP1 can associate with NRF2, promoting NRF2 degradation, which also reduces the p62 level. In addition, CYLD stimulation will contribute to p62 reduction through its autophagic degradation. Altogether, reduced p62 levels can lead to the inhibition of NRF2- and NF-kB-mediated transcription, creating a positive cycle (Katsuragi et al. 2016) (Fig. 1B).

**Fig. 1:**
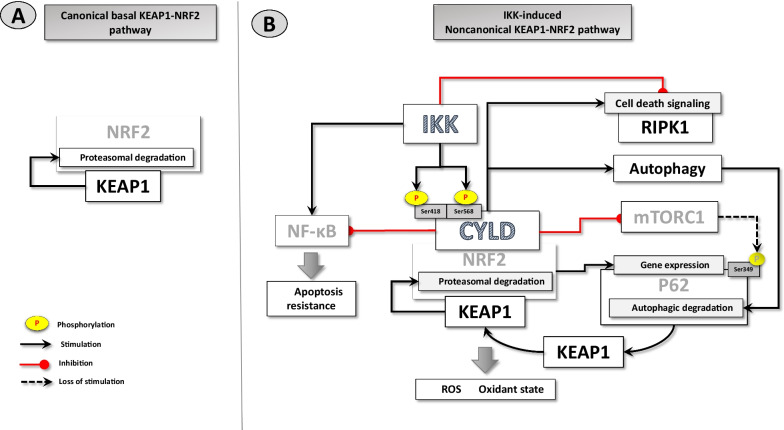
IKK-CYLD-NRF2 interaction. (**A**): In the resting cell, a low NRF2 level is present due to the canonical KEAP1-NRF2 interaction and degradation. (**B**): Various stimuli that activate the IKK signaling pathway also cause mTORC1 activation and autophagy inhibition. mTORC1 reduces NRF2 degradation by phosphorylating p62 scaffold protein, enhancing the p62-KEAP-1 interaction. However, during the terminal phase of signaling, IKK-mediated phosphorylation-dependent deconjugating activity of CYLD inhibits mTORC1, while stimulating the autophagic pathway. Thus, the resultant mTORC1 inhibition and a decrease in p62 level due to the autophagic degradation results in the loss of the p62-KEAP1 relationship. The liberated KEAP1 degrades NRF2 and inhibits its transcription activity

To summarize the results of IKK-stimulated CYLD activity: (1) decreases the affinity of p62 for KEAP1; thus, increases proteasomal degradation of NRF2 through the potentiated NRF2-KEAP1 association. This, in turn, will reduce the cellular redox potential, promoting oxidative stress and a reduction in p62 level as well; (2) increases autophagy, leading to the further p62 reduction; (3) stimulates RIPK1-mediated cell death (apoptosis and/or necroptosis); (4) inhibits apoptosis resistance, potentiating the RIPK1-mediated cell death.

ROS production was thought to be of key importance in ORN. NRF2 was generally thought to be a crucial cellular defense against oxidative stress (Chen et al. 2021). The findings of Chen et al., regarding NRF2 inhibition by IKK in ORN, which leads to increased oxidative stress, are important. However, their interpretation of the CLYD-NRF2 interaction does not seem to be compatible with the available data. This letter aims to reconcile conflicting results regarding the mechanism of NRF2 inhibition. Thus, it may make more sense for Chen et al. to use the model defined here, rather than their confusing implications, to explain the influence of CYLD on NRF2. This model can also be considered as a working mechanism for other chronic degenerative diseases in which IKK activation and oxidative stress are common denominators.

## Abbreviations

CYLD: Cylindromatosis protein
IKK: IκB kinase
KEAP1: Kelch-like ECH-associated protein 1
mTORC1: Mechanistic target of rapamycin
NF-κB: Nuclear Factor Kappa B
NRF2: NF-E2-related factor 2
ORN: Obesity-related nephropathy
ox-LDL: Oxidized low-density lipoprotein
RIPK1: Receptor-interacting protein kinase 1
ROS: Reactive oxygen species
